# Visual Maps Development: Reconsidering the Role of Retinal Efnas and Basic Principle of Map Alignment

**DOI:** 10.3389/fncel.2018.00077

**Published:** 2018-03-21

**Authors:** Elise Savier, Michael Reber

**Affiliations:** ^1^Centre National de la Recherche Scientifique, UPR3212 - Institute of Cellular and Integrative Neurosciences, University of Strasbourg, Strasbourg, France; ^2^Neuroscience, Department of Biology, University of Virginia, Charlottesville, VA, United States; ^3^Donald K. Johnson Eye Institute, Krembil Research Institute, University Health Network, Toronto, ON, Canada

**Keywords:** EphA, ephrin-As, retina, superior colliculus, visual cortex, visual mapping

## Introduction

**Figure 1:**
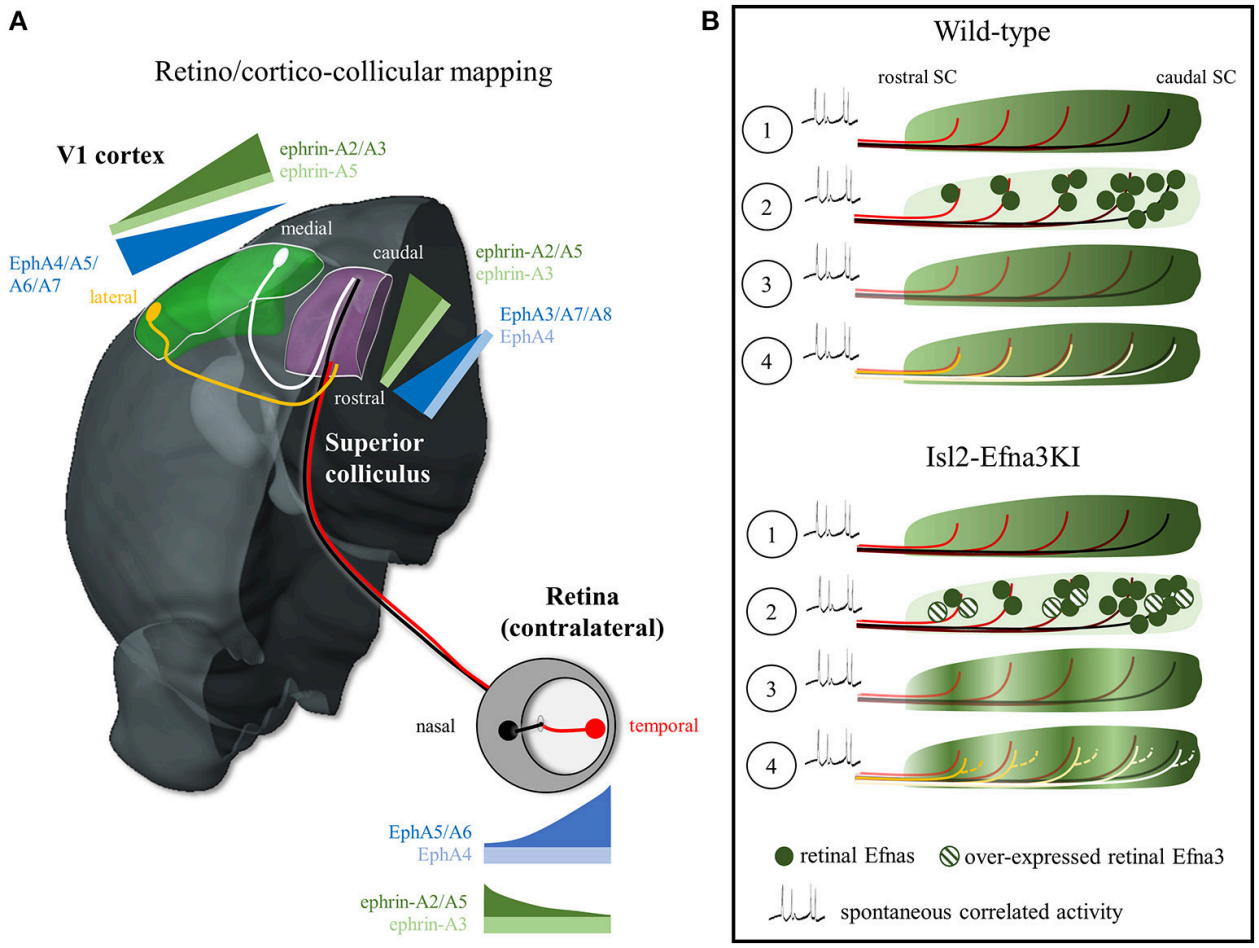
EphA/Efna expression, retino/cortico-collicular projection pattern and map alignment mechanism. **(A)** Representation of the EphA/Efna gradients of expression within the retino/cortico-collicular projection system in mouse. In the retina, temporal axons (red) expressing high levels of EphA and low levels of Efnas project in the rostral superior colliculus whereas nasal axons (black, low EphAs, high Efnas) project onto the rostral pole of the superior colliculus during the first postnatal week. From P6 to P12, V1 lateral axons (yellow, high EphAs) project to the superior colliculus and align with the temporal retinal projections. V1 medial axons (white, low EphAs) align with nasal retinal projections in the SC. **(B)** Map alignment principle. ① During retino-collicular projections, RGC fire spontaneous correlated activity and express EphA receptors on their axons that are engaged with collicular Efna gradients for mapping. ② During mapping, RGCs axons, carrying gradients of Efnas, transpose these ligands into the colliculus leading to ③ a smooth low-rostral to high-caudal gradient in Wild-type animals or to an oscillatory gradient in Isl2-Efna3KI animals. ④ Cortico-collicular projecting neurons, carrying EphA receptors, align with the retino-collicular map based on spontaneous correlated activity and V1 EphAs -> transposed retinal Efnas repulsive interactions. In Wild-type, V1 axons are facing a smooth gradient of transposed retinal Efnas leading to a linear homogeneous cortico-collicular map. In Isl2-Efna3KI animals, V1 axons are facing an oscillatory retinal Efnas gradient, leading to local duplications of the cortico-collicular map. SC, superior colliculus; V1, primary visual cortex.

Understanding and modeling the formation of neural maps has been a challenging subject in neurobiology, particularly in the visual system. One of the most studied visual mapping model corresponds to the topographic organization of the visual inputs in the superior colliculus -SC (or tectum in non-mammalian vertebrates), a layered structure in the midbrain involved in multisensory processing, controlling visual orientation and attention (May, 2006; Krauzlis et al., 2013). The superficial layers of the SC (*stratum griseum superficiale*-SGS and *stratum opticum*) receive organized inputs from ganglion cells in the retina (RGCs) and from neurons in layer V of the primary visual cortex (V1). RGCs extend their axons during embryonic life, forming the optic nerve, and organize in a retino-collicular map in the SC during the first postnatal week. Around post-natal day 6, while retino-collicular mapping is underway, axons from layer V neurons in V1 reach the SC, forming the cortico-collicular map, which aligns with the retino-collicular map in the SGS, so that corresponding sources of visual inputs are mapped topographically and adjusted with respect to the visual field: retinal nasal-temporal and V1 medial-lateral axes project onto the anterior (rostral)-posterior (caudal) axis in the SC (Figure 1A) and dorsal-ventral/anterior-posterior axes in retina/V1 project onto the lateral-medial axis of the SC (Cang and Feldheim, 2013). Evidence suggests that both the retino- and cortico-collicular inputs converge on the same cells in the SC and that spontaneous correlated activity in the retina during development instructs retino/cortico-collicular maps alignment (Boka et al., 2006; Triplett et al., 2009; Phillips et al., 2011). However, the molecular cues, if any, involved in the alignment of the visual maps in the SC are still unknown.

Potential candidates are the Eph tyrosine kinase receptors and their membrane-bound ligands, the Efns (ephrins), key players in the formation of visual maps (Flanagan and Vanderhaeghen, 1998; Lemke and Reber, 2005; McLaughlin and O'Leary, 2005; Scicolone et al., 2009; Triplett and Feldheim, 2012; Cang and Feldheim, 2013). They are divided in two sub-families, the EphB/Efnbs and the EphA/Efnas. Receptors and ligands are expressed in complementary gradients between interconnected structures (i.e., retina and SC/tectum or V1 and SC/tectum) and as reciprocal gradients within a structure (i.e., retina). Here we will focus on the EphA/Efna signaling system. During mapping, in mouse RGCs, EphA4/5/6 run from low-nasal to high-temporal, whereas Efna2/3/5 are expressed from high-nasal to low-temporal. In the SC EphA3/4/7/8 run from high-rostral to low-caudal and in V1 EphA4/5/6/7 show a high-lateral to low-medial gradient. Efna2/3/5 present a graded rostral low-caudal high expression in the SC and a lateral low-medial high gradient in V1 (Figure 1A; Park et al., 1997; Feldheim et al., 1998; Reber et al., 2004; Cang et al., 2005; Rashid et al., 2005). Most of the work addressing the role of EphA/Efna signaling in visual map formation focused on the activation of retinal EphAs by collicular Efnas (forward signaling). However clear answers about the function of the countergradients (retinal Efnas and collicular EphAs) have remained elusive.

## Hypotheses of the role of retinal efnas in retino-collicular mapping

Several hypotheses have been raised concerning the role of retinal Efnas. *In vitro* and *in vivo* evidence in chick suggested that Efnas present on RGCs modulate the activity of the retinal EphAs through interactions triggering phosphorylation of the receptor tyrosine kinase domain of the receptor or by silencing of the EphAs through inhibition of tyrosine phosphorylation. This mechanism generates a desensitization/masking of EphA receptors toward trans-binding of the Efna ligands in the SC/tectum, therefore sharpening the functional EphA gradients in the RGCs (Connor et al., 1998; Dütting et al., 1999; Hornberger et al., 1999; Carvalho et al., 2006). However, it is not clear whether EphAs desensitization in the retina occurs by cis-interaction, in the same RGCs or by trans-interaction, between neighboring RGCs in the retina/optic nerve, or both (Weth et al., 2014). Another hypothesis, based on *in vitro* and *in vivo* experiments in chick and mouse, suggested that retinal Efnas directly participate to the mapping process in the SC. Retinal Efnas are activated by collicular EphAs leading to axon branching inhibition posterior and anterior to the correct location of the termination zone in the SC (Yates et al., 2001; Rashid et al., 2005; Lim et al., 2008; Yoo et al., 2011).

## Limitations of previous experimental approaches

As summarized above, strong evidence from *in vitro* and *in vivo* analyses in chick and mouse unambiguously demonstrated the role of EphA/Efna signaling in retino-collicular mapping. However, their mechanism of action has remained controversial for several reasons. First, their presence in both projecting and target structures only allowed the characterization of their function at a system level in null-animals, precluding the assessment of the relative contribution of collicular, retinal and cortical gradients. Second, the co-expression of several members of both receptors and ligands made almost impossible the identification of a member-specific function. Finally, EphAs and Efnas are expressed as gradients, suggesting that growth cones detect variations of EphA/Efna concentrations over a given distance, indicating a quantitative mechanism (Goodhill, 1998; Goodhill and Baier, 1998). The homogeneous removal of EphAs or Efnas, as performed in knock-out approaches, provides qualitative information -whether this molecule is involved in mapping- but will not tell *how* it works. To address such a mechanism, a *quantitative* perturbation of the gradient should be performed by, for example, changing the orientation or the periodicity.

## New approaches for better insights into mapping mechanisms

In 2000, Brown and colleagues circumvent these constraints by developing an original gain-of-function approach, the Isl2-EphA3 knock-in mouse model (Isl2-EphA3KI), which presents a quantitative perturbation of the EphA receptor gradients in the retina (Brown et al., 2000). Such approach revealed to be extraordinarily informative in addressing the mechanism of action of the retinal EphAs because the perturbation of their expression occurred in the RGCs *only* (cell-specificity) and because the graded expression of EphAs was *quantitatively* altered, from a smooth to an oscillating gradient (change in periodicity). This approach, and subsequent work using the Isl2-EphA3KI model, yielded crucial findings about the mechanisms of retino-collicular and cortico-collicular mapping which have been impacting both experimental (Brown et al., 2000; Reber et al., 2004; Triplett et al., 2009; Bevins et al., 2011; Owens et al., 2015) and theoretical neurosciences (Koulakov and Tsigankov, 2004; Reber et al., 2004; Tsigankov and Koulakov, 2006, 2010; Willshaw, 2006; Grimbert and Cang, 2012; Sterratt, 2013; Sterratt and Hjorth, 2013; Hjorth et al., 2015; Owens et al., 2015). It is now widely acknowledged that *competition* and *relative EphA signaling* between RGCs control early steps of retino-collicular map formation.

Recently, tissue/cell-specific *in vivo* approaches were developed to address the role of retinal Efnas. Work from Drescher's lab, using a conditional deletion of retinal Efna5, suggested that the high expression of Efna5 on nasal RGC axons repels temporal RGCs axons, expressing high levels of EphAs, therefore preventing them to invade the caudal SC (Suetterlin and Drescher, 2014). This principle is based on a target-independent axon-axon interaction mechanism between RGCs, originally hypothesized by Bonhoeffer (Bonhoeffer and Huf, 1985; Weth et al., 2014). In a work recently published (Savier et al., 2017) we analyzed visual maps formation and alignment in the SC in Isl2-Efna3KI animals. Similar to the Isl2-EphA3KI model, the Isl2-Efna3KI mouse over-express the Efna3 ligand in 50% of the RGCs *only*, therefore creating an oscillatory gradient of Efnas in the RGCs (cell-specificity and quantitative perturbation). The analysis of the retino-collicular mapping in these animals showed no targeting perturbation, suggesting that Efna3 is not directly involved in retino-collicular mapping (Savier et al., 2017), further confirming results obtained from Efna3^−/−^ animals (Pfeiffenberger et al., 2006). Moreover, our results also suggested that the axon-axon interaction principle (Suetterlin and Drescher, 2014; Weth et al., 2014) does not seem to apply to Efna3, raising the question of member-specific function of Efnas in the visual system. Unlike the retinal EphAs (Bevins et al., 2011), the retinal Efnas do not seem to be functionally interchangeable.

A surprising observation was made when we analyzed cortico-collicular mapping in the Isl2-Efna3KI animals. In ~50% of the heterozygous and homozygous mutants, the cortico-collicular map was disrupted, presenting local duplications of the projections from V1 generating a misalignment with the retino-collicular map. This was confirmed when genetic inactivation of the over-expressed retinal Efna3 in the Isl2-Efna3KI mutants restored a wild-type cortico-collicular map (Savier et al., 2017). Although unexpected, this result is in line with previous studies demonstrating that retinal inputs are required for cortico-collicular map formation and alignment (Khachab and Bruce, 1999; Triplett et al., 2009) however, the mechanisms were unclear.

## Mechanistic models of retino/cortico-collicular maps alignment in the SC

Two alternative hypotheses have been suggested to account for the alignment of the cortico-collicular projections onto the retino-collicular map: a gradient-matching model and a retinal-matching model (Cang and Feldheim, 2013). The gradient-matching hypothesis suggests that V1 axons carrying EphAs are activated by collicular Efnas leading to the formation and alignment of the cortico-collicular map through forward signaling. Although forward EphA signaling is relevant, positional information provided by *collicular Efnas* to V1 ingrowing axons seems unlikely regarding the results obtained with the Isl2-EphA3KI and Isl2-Efna3KI models. In Isl2-EphA3KI, the collicular expression of Efnas is unaltered; however, a duplication of the cortico-collicular projections can be observed ruling out the gradient-matching model (Triplett et al., 2009). Further refutation of this model came from the results observed in the Isl2-Efna3KI mouse. A duplication of the cortico-collicular projections is observed in 50% of the mutants whereas the collicular expression of Efnas is normal (Savier et al., 2017).

What is the direct evidence supporting a retinal-matching model and what are the molecular players? A first indication came from work in anophthalmic mice that showed dispersed organization of the cortico-collicular projections suggesting that retinal inputs are required for the establishment of a precise topographic organization (Khachab and Bruce, 1999). This was confirmed in enucleated and Math5-null mice (lacking 90–95% of the RGCs) which showed enlarged cortico-collicular termination zones (Triplett et al., 2009). In the Isl2-EphA3 homozygote KI, both retino- and cortico-collicular maps are *fully duplicated but aligned* (Triplett et al., 2009). The colllicular duplication of the V1 projections copies the duplication of the retino-collicular map. Triplett and collaborators demonstrated that this duplication is due to correlated spontaneous activity, a stochastic process originating in the retina and propagating to the SC, which instructs the alignment of the cortico-collicular projections onto the retino-collicular map (Triplett et al., 2009). Further confirmation of the retinal-matching model and identification of the molecular effectors came from our analysis of the Isl2-Efna3KI animals (Savier et al., 2017). Here, the oscillatory expression of retinal Efna3, due to the over-expressed Efna3 in 50% of the RGCs, is transposed to the SC during retino-collicular mapping and provides positional information for incoming V1 axons carrying EphAs. The presence of an oscillatory Efna3 expression of retinal origin in the SC generates local duplications of V1 projections (Figure 1B). However, Efna3 by itself cannot provide positional information as it is not graded. Therefore, together with Efna3, other Efnas *presenting a graded expression* must be involved in cortico-collicular map alignment in the SC. Retinal Efna2 and Efna5 are good candidates and computational modeling supports this hypothesis (Savier et al., 2017) although some controversies appeared as nasal Efna5 has been shown to be involved in axon-axon interaction during retino-collicular mapping (Suetterlin and Drescher, 2014). Residual endogenous collicular Efnas, previously engaged during retino-collicular mapping, could also participate in the alignment of the V1 projections, although it has been shown that upon binding to EphAs, the EphA/Efna complexes are cleaved and internalized, making EphAs and Efnas unavailable for further guidance (Janes et al., 2005; Yoo et al., 2011).

Altogether, these results support a retinal-matching model for the alignment of the retino- and cortico-collicular maps. A recent view favors a mechanism based on spontaneous correlated activity (Triplett et al., 2009) and (partial) Efnas transposition from the RGCs into the SC during retino-collicular mapping (Savier et al., 2017). These Efnas of retinal origin then provide positional information, through forward signaling, to the ingrowing V1 axons bearing EphAs (Figure 1B, Movie 1). The variable penetrance of the cortico-collicular duplication observed in Isl2-Efna3KI mutants is likely to be the consequence of the stochastic nature of the mapping process where spontaneous correlated activity tends to associate neighboring projections in the SC whereas local oscillatory Efna3 tends to separate them through repulsion (Triplett et al., 2009; Savier et al., 2017).

## Conclusion

New genetic approaches recently challenged previous hypotheses about the role of retinal Efnas in visual map formation and uncovered novel principle and mechanism. This new concept suggests that a leading sensory map (the retino-collicular map) carries molecular cues (retinal Efnas) allowing subsequent sensory map alignment (cortico-collicular map) (Movie 1). In support of this concept, recent evidence suggests that sonic hedgehog is transported by contra-lateral RCGs axons to the optic chiasm to repel ipsi-lateral RGC axons (Peng et al., 2018). Such a principle could be relevant for sensory map formation allowing fine adjustments compensating for intrinsic variability. A similar process has been shown in the lateral geniculate nucleus, where V1 cortical inputs are required for the proper targeting of RCGs (Shanks et al., 2016). However, such new principle needs to be further challenged both experimentally and theoretically using appropriate animal models presenting tissue/cell-specific rearrangement.

## Author contributions

ES and MR wrote and edited the manuscript and designed the figure and the movie.

### Conflict of interest statement

The authors declare that the research was conducted in the absence of any commercial or financial relationships that could be construed as a potential conflict of interest.
